# Analysis of antibiotics consumption pattern among hospitalized patients in Nepal: a nationally representative multi-hospital survey

**DOI:** 10.1186/s12879-025-12187-5

**Published:** 2025-11-22

**Authors:** Elina Khatri, Pramod Joshi, Megha Raj Banjara, Meghnath Dhimal

**Affiliations:** 1 Nepal Health Research Council, Ramshah Path, Kathmandu, Nepal; 2 Central Department of Microbiology, Tribhuvan University, Kathmandu, Nepal

**Keywords:** Referral hospitals, Nepal, Antibiotics use, Inpatients

## Abstract

**Background:**

Antimicrobial resistance (AMR) is a global public health issue with multisectoral contribution. It increases morbidity, mortality and health related expenditures. Antibiotics are divided into three groups of Access, Watch and Reserve (AWaRe) in order to consider antibiotic stewardship. This study aimed at assessing the consumption pattern of AWaRe groups of antibiotics by inpatients in different hospitals of Nepal.

**Methods:**

This was a cross-sectional hospital based study. A proforma was developed to obtain data regarding the use of AWaRe groups of antibiotics. All the patients admitted to medical and surgical wards of the selected referral hospitals of seven provinces were included in the study and data on antibiotics use were collected for a month. Data was entered in Microsoft Excel and analyzed in IBM SPSS version 21 using descriptive statistics.

**Results:**

The consumption of AWaRe group of antibiotics among the inpatients were found to be 29.8%, 70.1% and 0.1% respectively. The most frequently consumed antibiotics were Metronidazole and Ornidazole for Access group, Ceftriaxone and Piperacillin tazobactam for Watch group and Linezolid for Reserve group.

**Conclusion:**

The target of increasing the proportion of global consumption of Access antibiotics to at least 60% of total consumption is stated by WHO. However, the minimal use of antibiotics from “Access” category and over use of antibiotics from “Watch” category can promote antimicrobial resistance. Rationale and appropriate use of antibiotics are important to minimize antimicrobial resistance.

## Background

Antibiotics are being used widely in the form of therapeutics treatment, prophylaxis and growth promoters among both human and animals [1]. The over and misuse of antimicrobials in human, animal and plant is considered as vital for developing drug resistant by pathogens [2]. Antibiotics consumption is found to be higher in health facilities of Nepal contributing to increased risk of inappropriate use of antibiotics [3]. Antimicrobial resistance (AMR) develops when pathogens stop responding to medications, making it more difficult to cure the illnesses. This increases the possibility of serious illness, death, and disease transmission. Antibiotic resistance in bacteria is particularly problematic due to its rapid rate of developing resistance to many new antibiotics and spread [4]. 

AMR is a silent and rapidly escalating pandemic, presenting a critical challenge to global health security including achievement of Universal Health Coverage and Sustainable Development Goals [5], the predictions are 10 million deaths annually by 2050 [6, 7]. The health care costs would rise up to 25% in low income countries and 8% globally due to AMR and its effects go beyond the health sector [8, 9]. According to World Bank, the global economy will lose nearly 4% of its annual Gross Domestic Product (GDP) by 2050 due to AMR with the severity of losses being greater in low- and middle-income countries (LMICs) [6]. Similarly, up to 28 million people, majority from developing nations may get forced into poverty by 2050 due to AMR and its impact on economic productivity, livestock production and health care costs [7]. 

The emergence and spread of AMR have been accelerated by irrational and inappropriate use of antimicrobials in humans, animals and plants, inadequate infection prevention and control (IPC) measures and a lack of equitable access to affordable and quality-assured antimicrobials [7]. Although antibiotic therapy is essential for the treatment of infections, prescribing them empirically prior susceptibility testing has led to increase in antibiotic resistance [10]. In Nepal, irrational antimicrobials use is common [8]. There is unnecessary prescription of more than one antibiotic to the patient by the pharmacies, without bacterial confirmation and susceptibility testing [9]. Self-medication is also common in Nepal, and most people do not comply with the physician prescribed duration or course of antibiotics treatment [11]. 

In order to rationalize the use of antimicrobials, the World Health Organization (WHO) updated the Essential Medicine List (EML) and categorized the antibiotics into three groups- Access, Watch and Reserve (AWaRe) in 2017. The WHO Expert Committee on the Selection and Use of Essential Medicines developed AWaRe classification of antibiotics as a tool to support antibiotic stewardship efforts at local, national, and global levels [12]. WHO had launched the Global Antimicrobial Resistance and Use Surveillance System (GLASS) to accomplish the goal of the Global Action Plan for managing AMR [13]. 

The national level data on antibiotic consumption are vital to track the current AMR trend and formulate AMR containment strategies based on evidence based approach [14]. However, there is no national level data analysis on antibiotics consumption by inpatients till date. There were few studies focused on antibiotic consumption performed on smaller scale at fewer health care settings. So, this study analyzed antibiotic consumption pattern by inpatients admitted to medical and surgical wards of the selected referral hospitals of seven provinces of Nepal. This study could serve as a foundation information from Nepal on antibiotics consumption among inpatients of the hospitals.

## Methods

### Study design

**Fig. 1:**
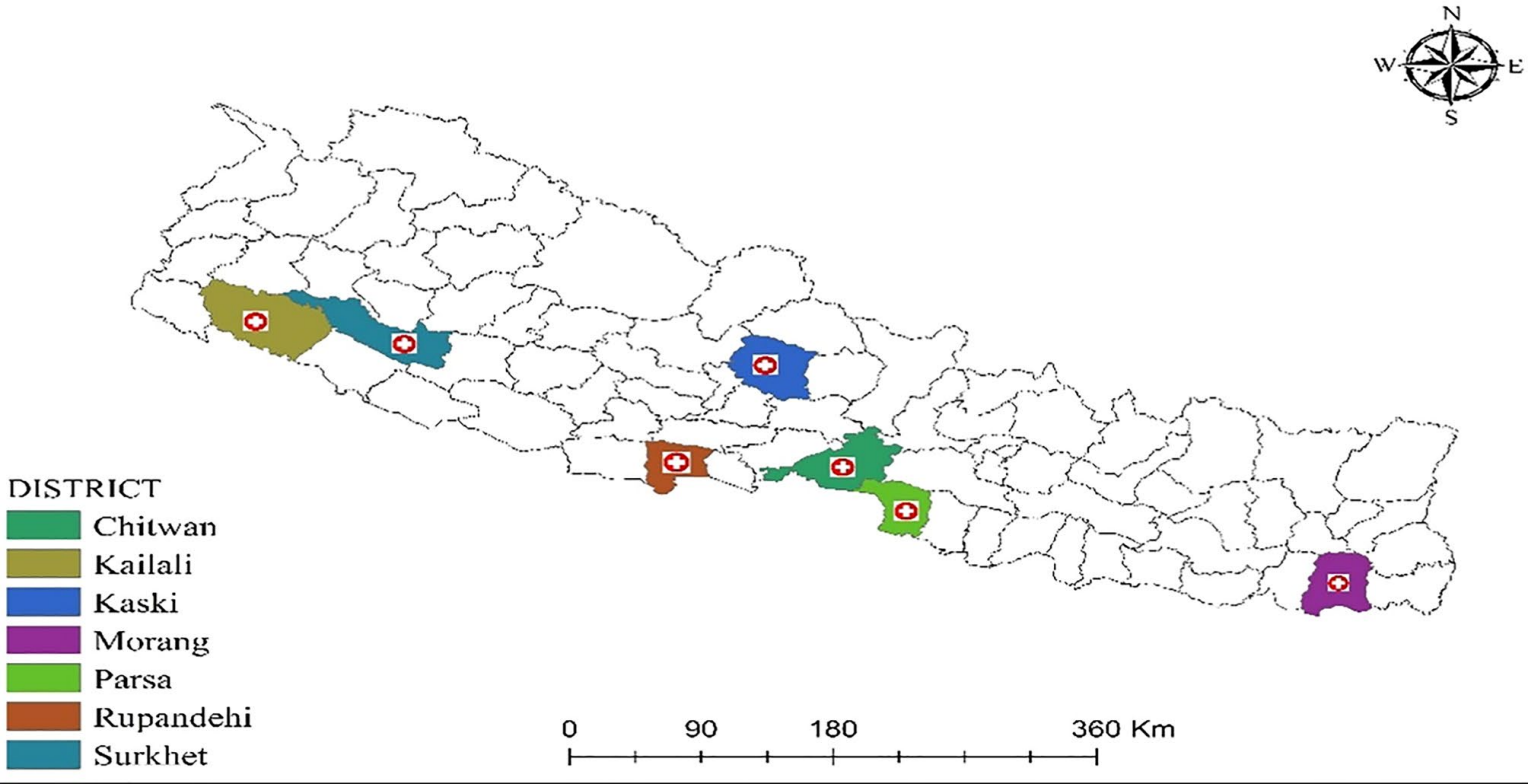
Map showing study sites of each province

This was a hospital based cross-sectional study conducted in country wide representative samples of hospitals of all seven provinces of Nepal. Hospitals were selected purposively to ensure representation from each province and sector **(**Fig. 1**)**.

### Study setting

Nepal has a mixed healthcare system comprising of both public and private health institutions that deliver varying levels of care across the administrative areas [15]. One public and one private referral hospitals from each of the seven provinces were selected for the study **(**Table 1**)**. A province wise list of both public and private hospitals was prepared. One public and one private hospital per province with highest number of bed capacity in the province were selected. Among seven public hospitals, the bed capacity ranged from 125 to 750 whereas among seven private hospitals the bed capacity ranged from 150 to 900. These hospitals served for most of the outpatient and in-patients annually in the provinces. These hospitals were tertiary care referral level hospitals, located in province headquarters and major cities.

**Table 1:** List of selected hospitals

S.*N*.	Province	Type	Name of hospitals
1.	Koshi	Public	Koshi Hospital
		Private	Nobel Medical College Teaching Hospital Ltd.
3.	Madhesh	Public	Narayani Hospital
		Private	National Medical College Teaching Hospital
5.	Bagmati	Public	Bharatpur Hospital
		Private	Chitwan Medical College and Teaching Hospital
7.	Gandaki	Public	Western Regional Hospital
		Private	Gandaki Medical College Teaching Hospital & Research Centre (P) LTD.
9.	Lumbini	Public	Lumbini Provincial Hospital
		Private	Nepalgunj Medical College
11.	Karnali	Public	Province Hospital, Karnali
		Private	Karnali Care International Hospital and Research Center Pvt. Ltd
13.	Sudhurpaschim	Public	Seti Provincial Hospital
		Private	Nisarga Hospital & Research Center Pvt. Ltd

### Participants

All the patients admitted to medical and surgical wards of the selected referral hospitals were included in the study. Antibiotics consumed by inpatients in medical and surgical wards of the hospitals in one-month period were recorded during June to July, 2022. In this study, the information on antibiotics use were recorded from altogether 3227 inpatients for a duration of one month.

### Measures

All inpatients admitted to medical and surgical wards of selected referral hospital with ongoing treatment with antibiotics were included in the study. Inpatients of both gender and age above 18 years were included. Patients below 18 years of age were not included in the study as the criteria of calculating Daily Defined Dose (DDD) varied between pediatric and adult patients. Patients given antibiotics by different routes of administration like oral, parenteral, rectal or through inhalation were also included.

The proforma was developed to obtain information regarding the antibiotics used, its doses, route of administration and number of hospital stay of the patients. The approvals were obtained from all the selected hospitals prior data collection. One nursing staff from each ward was selected and trained about the data collection procedure prior initiating the collection process. The details about the attributes included in the proforma and its relevancy in calculating the Daily Defined Dose (DDD) for each antibiotics was well explained to them. Then after, the trained nurses reviewed patients’ records to collect information on the dose of antibiotics, their routes, frequency of administration and duration of their use until the last day of admission.

### Ethical considerations

The study protocol was approved from the Ethical Review Board of Nepal Health Research Council (Registration no: 585/2021). Data on antibiotics consumption were obtained from the hospital records for each admitted patients. Patients were not directly involved or interviewed; therefore, individual informed consent was not required. The approval for conduction of the study was obtained from the hospital administration prior data collection. The data was kept confidential and no personal identifiers were used while analyzing data and writing the report.

### Statistical analysis

The collected data was entered in Microsoft Excel and analyzed using IBM SPSS version 21. The antibiotics consumed by the inpatients was computed in terms of Daily Defined Doses (DDD) for each antibiotic. The WHO AWaRe framework and the 2021 Anatomical Therapeutic Classification (ATC) system was used to evaluate antibiotic use patterns as listed in the WHO Model List of Essential Medicine [16, 17]. The Daily Defined Doses (DDD) were calculated by multiplying the quantity field by DDD conversion factor field on the 2022 version of the ATC/DDD index using the formula DDD/100 bed days = Number of units administered in a given period (milligram) ×100/DDD (milligram)×number of days in the period×number of beds×occupancy index [17]. A bed-day was defined as overnight stay in hospital. The consumption pattern was calculated based on categories of antibiotics, type of ward, and mean DDDs.

## Results

### Consumption of antibiotics based on AWaRe classification

**Fig. 2:**
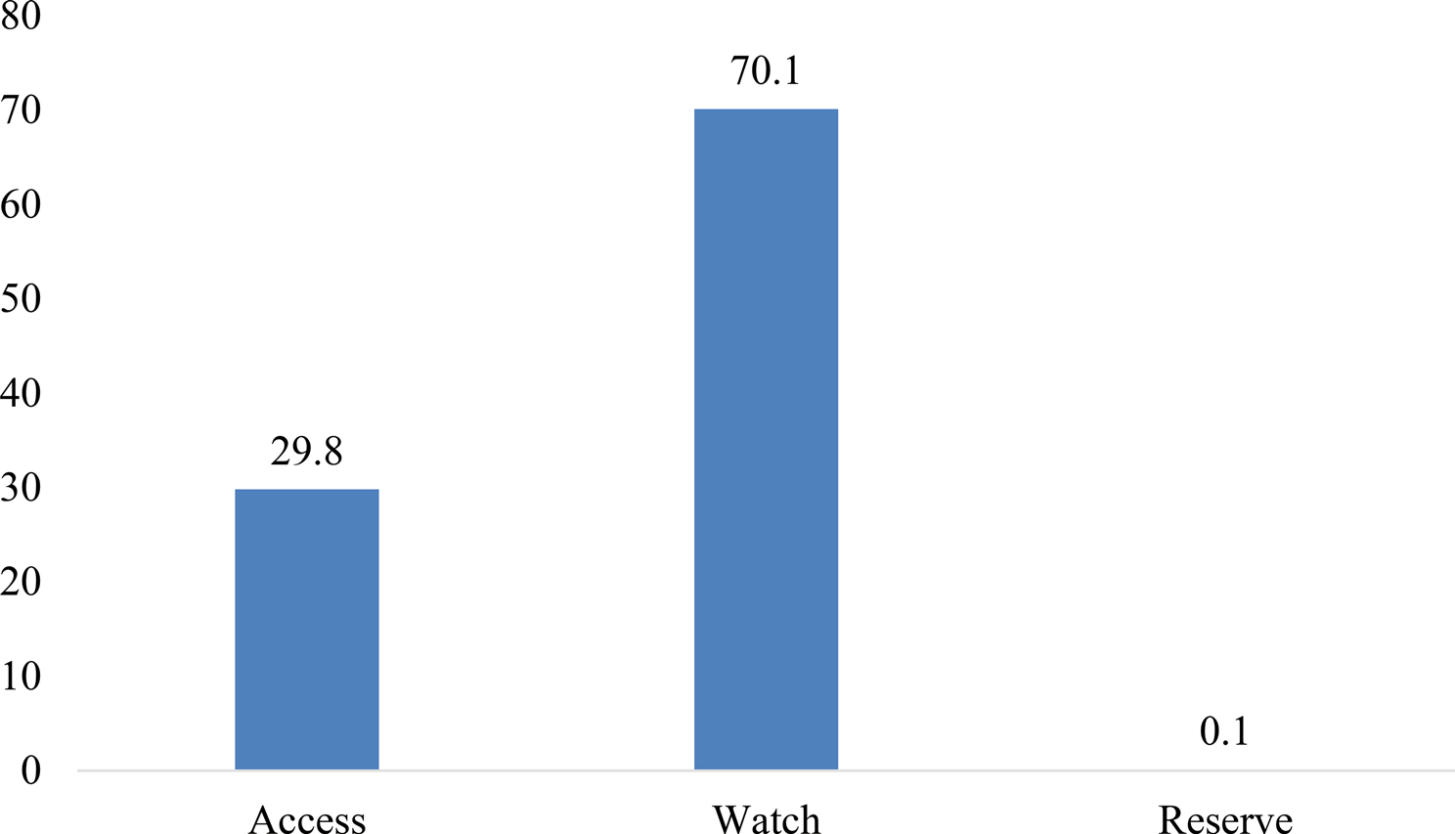
Consumption of AWaRe class of antibiotics

The majority of antibiotics consumed were from the watch group (70.1%) followed by access group (29.8%) and reserve group (0.1%) **(**Fig. 2**).**

The five most frequently consumed antibiotics in hospital inpatients were Ceftriaxone (34.4%), Metronidazole (9.2%), Piperacillin tazobactam (8.6%), Ornidazole (7.2%) and Levofloxacin (4.6%) respectively. It seems that diverse antibiotics have been prescribed for the patients **(**Table 2**).**

**Table 2:** Consumption of antibiotics based on AWaRe classification

Name of antibiotics	Number	Percent
**Access**		
Amikacin	91	1.8
Amoxycillin	188	3.6
Ampicillin	40	0.8
Ampicillin cloxacillin	18	0.3
Cefadroxil	2	0.0
Cefazoline	3	0.1
Clindamycin	101	1.9
Doxycycline	107	2.1
Flucloxacillin	112	2.2
Gentamycin	12	0.2
Metronidazole	477	9.2
Nitrofurantoin	11	0.2
Ornidazole	373	7.2
Secnidazole	1	0.0
Tetracycline	13	0.3
**Watch**		
Azithromycin	232	4.5
Cefepime	108	2.1
Cefixime	95	1.8
Cefoperazone	124	2.4
Cefotaxime	89	1.7
Cefpodoxime	9	0.2
Ceftazidime	3	0.1
Ceftriaxone	1791	34.4
Cefuroxime	91	1.8
Ciprofloxacin	86	1.7
Clarithomycin	14	0.3
Imipenem	6	0.1
Levofloxacin	237	4.6
Meropenem	86	1.7
Moxifloxacin	46	0.9
Norfloxacin	3	0.1
Ofloxacin	7	0.1
Piperacillin	136	2.6
Piperacillin tazobactam	446	8.6
Rifaximin	16	0.3
Tobramycin	5	0.1
Vancomycin	13	0.3
**Reserve**		
Linezolid	7	0.1
**Total**	**5199**	**100.0**

Similarly, the average number of DDDs and DDDs per 100 Occupied Bed Days was computed to measure the antibiotic consumption in hospitals in relation to patient bed days. The average number of DDDs for each antibiotic were found to be higher than the standard Daily Defined Dose (DDD) referred by WHO for each antibiotic. The average DDD for Azithromycin, Cefixime, Cefpodoxime, Ciprofloxacin, Clarithromycin and Doxycycline were found to be used in higher intensity than the reference stated by WHO (Table 3).

**Table 3:** Consumption based on DDDs per 100 OBDs

Name of antibiotics	WHO standard (DDD*)	Mean (DDD)	Mean (DDDs per 100 OBDs**)
Amikacin	1	3.7	66.8
Amoxycillin	3 (1.5)	5	101.5
Ampicillin	6	1.4	38.6
Ampicillin cloxacillin	8	1.3	41.6
Azithromycin	0.50	7	139.9
Cefadroxil	2	2	33.3
Cefazoline	3	1.7	61.1
Cefepime	4	3.6	75.9
Cefixime	0.40	4.1	78.7
Cefoperazone	4	2.5	66.9
Cefotaxime	4	4.1	69.2
Cefpodoxime	0.40	12.6	334.4
Ceftazidime	4	3.9	48.1
Ceftriaxone	2	3.6	85.6
Cefuroxime	0.50	4.1	81.5
Ciprofloxacin	0.80 (1)	2.8	64.0
Clarithomycin	0.5	12.2	159.6
Clindamycin	1.8 (1.2)	5.8	72.9
Doxycycline	0.10	10	196.4
Flucloxacillin	2	4.6	101.9
Gentamycin	0.24	2.8	54.8
Imipenem	2	4.4	49.6
Levofloxacin	0.50	6.4	102.1
Linezolid	1.20	4.8	51.9
Meropenem	3	5.9	73.3
Metronidazole	1.5 (2)	3.8	80.3
Moxifloxacin	0.40	5	94.6
Nitrofurantoin	0.20	5	84.1
Norfloxacin	0.80	3.5	83.3
Ofloxacin	0.40	4.5	126.2
Ornidazole	1 (1.5)	4.3	81.2
Piperacillin	14	4.9	80.1
Piperacillin tazobactam	14	4.9	79.6
Rifaximin	0.60	12.4	190.0
Secnidazole	2	0.50	25.0
Tetracycline	1	1.5	26.2
Tobramycin	0.24	2.7	42.6
Vancomycin	2	5.2	73.8

*DDD - Daily Defined Dose

**OBDs - Occupied Bed Days

### Ward-wise consumption of antibiotics

**Fig. 3:**
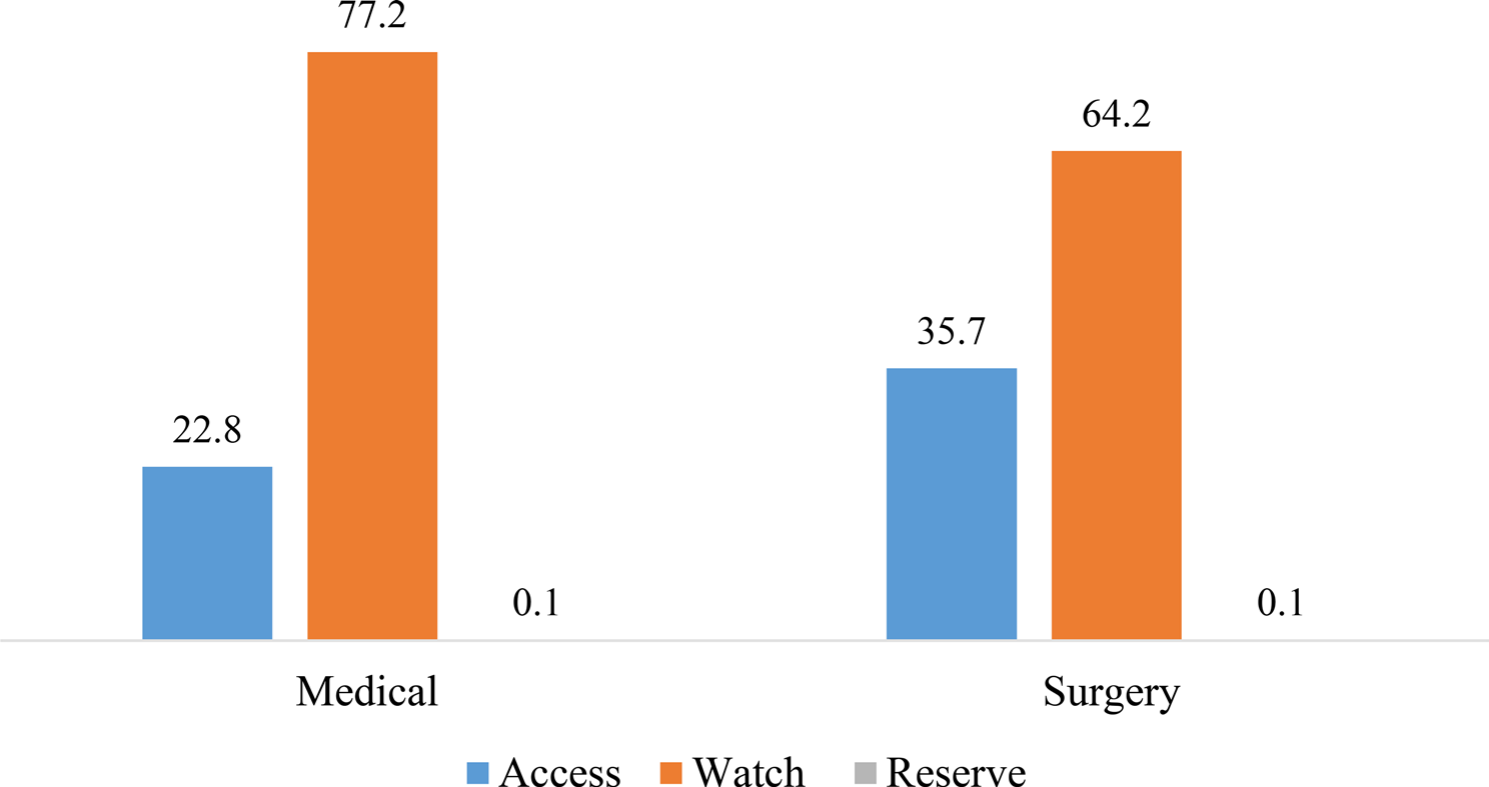
Consumption of antibiotics based on AWaRe category

The study found that both medical and surgical wards majorly i.e. 77.2% and 64.2% used antibiotics from Watch category. Likewise, 22.8% and 35.7% of antibiotics used in medical and surgical ward were from Access category (Fig. 3).

**Fig. 4:**
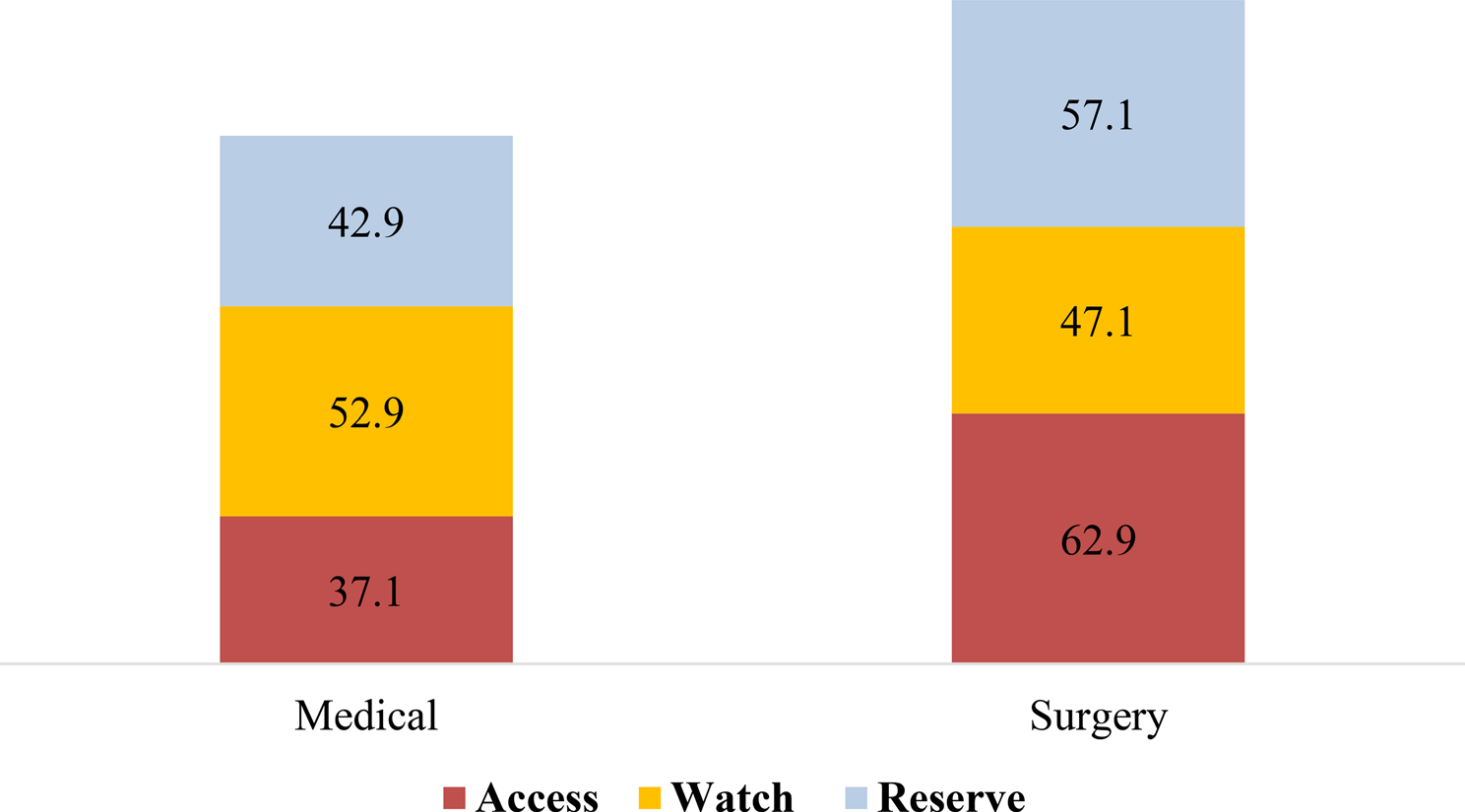
Consumption of antibiotics based on admitted ward

The study found that majority of antibiotics from Access (i.e. 62.9%) group were used among patients in surgical wards, whereas Watch (i.e. 52.9%) group were used among patients admitted to medical wards **(**Fig. 4**).**

## Discussion

This multi-hospital based study aimed at assessing the consumption pattern of AWaRe classification of antibiotics at national level and to our knowledge this is the first nationally representative study in Nepal. The rigorous data collected for a month for each inpatients was used to obtain the DDD for each category of antibiotics.

This study provides an overview of the magnitude of hospital based consumption of antibiotics in Nepal based on the AWaRe classification. Its strength is coverage of data from both public and private referral level hospitals in all seven provinces of Nepal. This serves as a nationally representative baseline survey on consumption of antibiotics. The notable findings of antibiotic consumption include- the share of Access group of antibiotics consumption was found to be very lower than WHO recommendation. Although, Government of Nepal has adopted various plan and policies towards antimicrobial stewardship, the achievement was not as per the target which clearly reflects the need of strict implementation of strategies for improving the pattern of antibiotic consumption.

In this study, the consumption of the antibiotics from access category (29.8%) was significantly lower than the watch category (70.1%), although the WHO recommendation is to use at least 60% of access category of antibiotics. This might be due to the nature of health care settings selected for the study as it is a tertiary level referral hospital where patients usually visit for complicated conditions. WHO has recommended that the share of antibiotics consumptions should be more from the narrow-spectrum access category to reduce the selection pressure associated with the broad-spectrum watch and reserve category [16]. Similar situations were reported from the study done in Sichuan Western China in 2020 [18]. The finding is similar with study done in India as well where consumption of watch group antibiotics (37%) is relatively higher than access group (17%) [19]. The report on Consumption and Rational Use of Antimicrobials in South-East Asia Region also clearly highlighted that Nepal exhibit elevated consumption of Watch category antibiotics [17]. The studies in future should focus on audit of the use of Watch group of antibiotics. This escalation in consumption pattern signifies the urgent need of targeted interventions and antimicrobial stewardship practices within the country. However, the Antimicrobial Stewardship Program (ASP) in Nepal has been limited with existing challenges such as lack of adequate knowledge and lower level acceptance from physicians, and limitations related to diagnostic testing and antibiotics choice [20] which needs to be addressed for better implementation of ASP in Nepal and achieve the WHO recommendation for antibiotic.

In this study, the top most consumed antibiotic was Ceftriaxone from the Watch group similar to the findings of previous studies conducted at Lalitpur district Nepal [21] and Sierra Leone [22]. We found only 0.1% of antibiotics consumed were from Reserve group similar to the findings of study done in Sichuan West China where it was only 0.07% [18]. Our and other studies indicate that antibiotic prescription should adhere to global recommendations optimizing antibiotic use in the hospitals.

We found that the top five most frequently consumed antibiotics were Ceftriaxone, Metronidazole, Piperacillin tazobactam, Ornidazole, and Levofloxacin respectively. These findings are comparable with findings related to prescription of antibiotics where Ceftriaxone, Amoxicillin plus Cloxacillin, Azithromycin, and Cefixime were among the most commonly prescribed antibiotics [3]. Ceftriaxone was among the top three most prescribed antibiotics in all the five clinical departments prescribing maximal antibiotics [3]. The findings is in contrast with study done in India [19] and China [18]. Cefoperazone-sulbactam and piperacillin-tazobactam were reported as the most frequently consumed antibiotics in India, whereas in China, Amoxicillin, Cefuroxime, Cefixime, Levofloxacin and Metronidazole were the most consumed antibiotics. Unlike this study, the consumption of Penicillin and Amoxicillin/beta-lactamase inhibitor was low in China, accounting for only 3.1% and 5.3% respectively [18]. This pattern of antibiotics prescription and consumption signifies the issues related to rationale prescription and use of antibiotics in Nepal and suggest for focused actions to prevent and control of AMR. The critical steps include follow of WHO AWaRe guidelines for antibiotic prescribing practices and regular audits of antibiotics use to ensure adherence to this guideline. Further, educating health care providers on AWaRe classification, promoting rational practices and following the evidence based practices are also important since study done among prescribers demonstrated inadequacy of knowledge regarding appropriate use of antibiotics [23]. These measures play important role in preserving the effectiveness of antibiotics within constant appearance of antibiotic resistance. 

This study was limited by the cross-sectional design and conducted in referral level hospitals for a month, so the results may not apply to other health care settings. Secondly, we do not follow up the individual patient’s behavior/practice towards antibiotic consumption beyond hospital settings in order to observe their actual practice regarding antibiotic use. Despite these limitations, this study provides valuable insights of antibiotic prescribing patterns of referral hospitals in Nepal.

## Conclusion

The antibiotics consumption by inpatients is highest from the watch group followed by access group highlighting the excessive and inappropriate prescriptions. Antibiotic stewardship program reinforcement, and surveillance of antibiotic consumption based on AWaRe classification, and adoption of AWaRe framework are needed to improve rationale antibiotic prescription for mitigating the risk of antibiotic resistance.

## Data Availability

The raw data supporting the conclusion of this article can be accessed from corresponding author upon reasonable request.

## Abbreviations

AMR: Antimicrobial Resistance
AST: Antimicrobial Stewardship Program
ATC: Anatomical Therapeutic Chemical
AWaRe: Access Watch Reserve
DDD: Daily Defined Dose
EML: Essential Medicine List
GDP: Gross Domestic Products
GLASS: Global Antimicrobial Resistance and Use Surveillance System
IBM: International Business Methods
IPC: Infection Prevention Control
LMICS: Lowe Middle Income Countries
OBD: Occupied Bed Days
SPSS: Statistical Package for the Social Sciences

## References

[1] Rijal KR, Banjara MR, Dhungel B, Kafle S, Gautam K, Ghimire B, et al. Use of antimicrobials and antimicrobial resistance in Nepal: a nationwide survey. Sci Rep 2021;11:1–14. 10.1038/s41598-021-90812-4. 10.1038/s41598-021-90812-4 PMC8172831 34078956

[2] World Health Organization. (2023) Antimicrobial Resistance. Available at: https://www.who.int/news-room/fact-sheets/detail/antimicrobial-resistance (last accessed November, 2025).

[3] Dixit SM, Bhatta S. Antibiotic prescribing pattern in different clinical departments at Kathmandu Medical College Teaching Hospital. J Kathmandu Med Coll 2018;7:18–25. 10.3126/jkmc.v7i1.20624.

[4] Prestinaci F, Pezzotti P, Pantosti A. Antimicrobial resistance: a global multifaceted phenomenon. Pathog Glob Health 2015;109:309–18. 10.1179/2047773215Y.0000000030. 10.1179/2047773215Y.0000000030 PMC4768623 26343252

[5] World Health Organization. (2019). WHO AWaRe classification database of antibiotics for evaluation and monitoring of use. Available at: https://www.who.int/publications/i/item/WHOEMPIAU2019.11 (Last accessed November 2025).

[6] World Organisation for Animal Health (OIE). (2021) Strategy on antimicrobial resistance and the prudent use of antimicrobials. Available at: https://www.woah.org/app/uploads/2021/03/en-amr-strategy (Last accessed November 2025).

[7] World Health Organization, the United Nations Sustainable Development Cooperation Framework. (2021). Antimicrobial resistance and. World Health Organization Report; 1–24. Available at: https://www.who.int/publications/i/item/9789240036024 (Last accessed November 2025).

[8] Nepal G, Bhatta S. Self-medication with antibiotics in WHO Southeast Asian Region: a systematic review. Cureus. 2018;10. 10.7759/cureus.2428 PMC5988199 29876150

[9] Acharya KP, Wilson RT. Antimicrobial resistance in Nepal. Front Med 2019;6:7–9. 10.3389/fmed.2019.00105. 10.3389/fmed.2019.00105 PMC6543766 31179281

[10] Huttner A, Harbarth S, Carlet J, Cosgrove S, Goossens H, Holmes A. Antimicrobial resistance: a global view from the 2013 World Healthcare-Associated Infections Forum. Antimicrob Resist Infect Control 2013;2:1–13. 10.1186/2047-2994-2-31. 10.1186/2047-2994-2-31 PMC4131211 24237856

[11] McEwen SA, Collignon PJ. Antimicrobial resistance: a one health perspective. Microbiol Spectr 2018;6. 10.1128/microbiolspec.arba-0009-2017. 10.1128/microbiolspec.arba-0009-2017 PMC11633550 29600770

[12] Jha N, Thapa B, Pathak SB, Kafle S, Mudvari A, Shankar PR. Availability of access, watch, and reserve (AWaRe) group of antibiotics in community pharmacies located close to a tertiary care hospital in Lalitpur, Nepal. PLoS ONE 2023;18:1–10. 10.1371/journal.pone.0294644. 10.1371/journal.pone.0294644 PMC10659150 37983218

[13] WHO Evaluation Office, Drew R. Comprehensive review of the WHO Global Action Plan on antimicrobial resistance. WHO Evaluation Office 2021;1:1-112. Available at: https://cdn.who.int/media/docs/default-source/documents/about-us/evaluation/gap-amr-final-report-v2.pdf (Last accessed November 2025).

[14] Poudyal N, Gallagher P, Joh HSJ, Prifti K, Eraly E, Chi K, et al. Final report: Nepal – capturing data on antimicrobial resistance patterns and trends in use in regions of Asia. Seoul (South Korea): International Vaccine Institute; 2022.

[15] Sah MK, Sangroula RK, Kumar A. Comparative analysis of performance of private and public healthcare systems in Nepal. Int J Community Med Public Health 2020;7:2462–2468. 10.18203/2394-6040.ijcmph20202966.

[16] Sharland M, Gandra S, Huttner B, Moja L, Pulcini C, Zeng M, et al. Encouraging AWaRe-ness and discouraging inappropriate antibiotic use: the new 2019 Essential Medicines List becomes a global antibiotic stewardship tool. Lancet Infect Dis 2019;19:1278–80. 10.1016/S1473-3099(19)30532-8 10.1016/S1473-3099(19)30532-8 31782385

[17] World Health Organization, Regional Office for South-East Asia. (2024). Consumption and rational use of antimicrobials in South-East Asia Region. Available at: https://www.who.int/publications/i/item/9789290211266 (last accessed November 2025).

[18] Song H, Liu X, Zou K, Li H, Fei H, Huang L, et al. Assessment of antibiotic consumption patterns in hospital and primary healthcare using WHO Access, Watch and Reserve Classification (AWaRe) in Sichuan Western China: 2020. Arch Public Health 2024;82:182. 10.1186/s13690-024-01391-5. 10.1186/s13690-024-01391-5 PMC11472543 39402638

[19] Ahmed MN, Thakur AK, Srivastava S, Ningombam A, Kirti M, Sagar S, et al. Assessment of antibiotic utilization patterns in an Indian Level-1 Trauma Center: a pilot study exploring days of antibiotic spectrum coverage and defined daily doses using WHO AWaRe classification trends. Front Antibiot 2025;4:1–8. 10.3389/frabi.2025.1578217. 10.3389/frabi.2025.1578217 PMC12304517 40735435

[20] Khanal S, Acharya U, Trotter AB, Tripathi P, Koirala S, Pahari B, et al. Challenges and opportunities in the implementation of an antimicrobial stewardship program in Nepal. Antimicrob Steward Healthc Epidemiol 2023;3:1–6. 10.1017/ash.2022.359.

[21] Baral P, Hann K, Pokhrel B, Koirala T, Thapa R, Bijukchhe SM, et al. Annual consumption of parenteral antibiotics in a tertiary hospital of Nepal, 2017–2019: a cross-sectional study. Public Health Action 2021;11:52–57. 10.5588/pha.21.0043. 10.5588/pha.21.0043 PMC8575388 34778016

[22] Lakoh S, Williams CEE, Sevalie S, Russell JBW, Conteh SK, Kanu JS, et al. Antibiotic use and consumption among medical patients of two hospitals in Sierra Leone: a descriptive report. BMC Infect Dis 2023;23:1–8. 10.1186/s12879-023-08517-0. 10.1186/s12879-023-08517-0 PMC10612171 37891476

[23] Sarraf DP, Rai D, Rauniar GP. Knowledge, attitude and practices on antibiotic use and resistance among doctors in B.P. Koirala Institute of Health Sciences. J Drug Delivery Ther 2018;8:170–5. https://jddtonline.info/index.php/jddt/article/view/1753.

